# The King's Last Illness

**Published:** 1910-05-14

**Authors:** 


### THE DEATH OF KING EDWARD VII.

### THE KING'S LAST ILLNESS.

His Majesty King Edward VII. passed peacefully away at 11.45 o'clock on Friday evening, May 6, in the presence of Queen Alexandra and their Royal Highnesses the Prince and Princess of Wales, the Princess Royal, and other members of the Royal Family.

The death of the King came with unexpected suddenness, although it was known that for some weeks his Majesty had been ailing. The story of his last illness, as told in the official bulletins, is meagre in the extreme, but amplified as these notices are by the semi-official announcements concerning his illness, the full record of his case is easily compiled. On crossing over to France, on his recent holiday, the late King suffered severely from dyspepsia, which obliged him to curtail one of his engagements while in Paris. He there visited a presentation of "Chantecleer," and on emerging from the theatre is stated to have caught a chill. An attack of bronchitis developed, which was accompanied with paroxysms of exhausting cough and some prostration. At that time, however, there was no heart trouble, his Majesty's pulse being good and his strength, in the circumstances, excellent. His medical advisers were therefore justified in contradicting the rumours that he was seriously ill, and to indulge in the hope that a sojourn at Biarritz would completely restore his health. Unfortunately the climatic conditions at the French health-resort were not ideal this April. It was impossible for his Majesty to be as much in the open air as possible, and during the latter part of his stay he was kept extremely busy by matters of State which needed his urgent attention. In consequence of the gravity of the political situation, his Mediterranean trip, which it is conceivable would have taken him out of the cold zone and reinvigorated him, had to be cancelled, and with the exception of short motor-trips into Spain his Majesty practically confined his holiday to Biarritz. There he shook off his attack of bronchitis, though he did not completely regain health and strength. On his return to London he threw himself with his characteristic ardour and unselfishness into his work, and this no doubt to a large extent contributed to his relapse. It was known that his Majesty was extremely subject to cold, and that his cough had to some extent become chronic. He was eager to visit his estate at Sandringham to inspect the recent alterations, and accordingly left London on the Friday before his last illness and spent part of the week-end at Sandringham. Here, notwithstanding the inclemency of the weather, he walked about and apparently caught another chill, which developed rapidly, so that on Monday afternoon his cough was harassing. No public notification was deemed necessary, the physicians-in-ordinary being justified in the hope that his Majesty would shake off the attack as he had done on the previous occasion. These hopes were, however, illusory, and on the Tuesday his Majesty's condition gave rise to serious alarm. His strength was obviously failing, but in spite of what his medical advisers counselled he persisted in transacting business of State, and worked with his secretaries at his bedside. On Wednesday his symptoms had not improved, his temperature being a degree above normal and his pulse rate, normally slow, 90, while he complained of laryngeal irritation. The public, who had reason to be alarmed owing to the rumours that were prevalent, was made aware of the seriousness of his case on Thursday, when the official bulletin, signed by the physicians-in-ordinary, was published. This bulletin was slightly modified by his Majesty himself. Further official notices, signed in addition by Drs. Dawson and St. Clair Thomson, were published on Friday, and kept the public informed of the full gravity of the situation, although it was apparent that few laymen realised the danger of what was generally taken to mean an ordinary attack of bronchitis. No definite information regarding the symptoms from which the King suffered was given in the bulletins, but it was reasonable to infer that the imminent danger to the Royal patient was cardiac failure. This was indeed the case, and his Majesty's death was similar to that of many elderly patients suffering from bronchitis and emphysema and succumbing ultimately to heart failure brought about by exhausting fits of coughing. The signs in his Majesty's case were perfectly clear, and there was no doubt about the diagnosis. There was no throat affection beyond the soreness and slight subacute laryngitis inevitable in such cases where an irritating cough exists, and in this case they were probably increased by the fact that his Majesty was an ardent smoker, whose laryngeal condition was in some degree similar to that which is usually known as smokers' throat. The increasing difficulty in breathing, during the last two days of his illness real attacks of dyspnœa, not only greatly exhausted the patient, but threw an immense strain upon an already overtaxed heart. In the circumstances the cause of death is perfectly clear, and it is unlikely that further details of medical importance regarding the King's last illness will be officially communicated to the public.

On Friday, the day on which he died, his Majesty continued to transact important and urgent business of State. He remained seated in an armchair, and saw his secretaries, though he received no visitors in audience as he had done on the Thursday. He found the greatest ease sitting in his chair, and refused to go to bed, although he fully realised the gravity of his condition. Later on in the day he became too weak to remain in his chair or to speak, and was removed to his bed. Shortly afterwards he became practically unconscious, and died two hours later.

